# Going beyond classic echo in aortic stenosis: left atrial mechanics, a new marker of severity

**DOI:** 10.1186/s12872-019-1204-2

**Published:** 2019-10-10

**Authors:** Patrícia Marques-Alves, Ana Vera Marinho, Rogério Teixeira, Rui Baptista, Graça Castro, Rui Martins, Lino Gonçalves

**Affiliations:** 1 Department of Cardiology, Centro Hospitalar e Universitário de Coimbra, Praceta Mota Pinto, 3000-001 Coimbra, Portugal; 2 iCBR, Coimbra Institute for Clinical and Biomedical Research, Universidade de Coimbra, Coimbra, Portugal; 3 Faculty of Medicine, Universidade de Coimbra, Coimbra, Portugal

**Keywords:** Aortic stenosis, atrial mechanics, two-dimensional speckle-tracking strain

## Abstract

**Background:**

There is limited information regarding left atrial (LA) mechanics in aortic valve stenosis (AS). We assessed LA mechanics in AS through speckle-tracking echocardiography (STE) according to severity and prognosis.

**Methods:**

We included 102 patients diagnosed with severe AS (sAS) and 80 patients with moderate AS (mAS), all with preserved ejection fraction and no coronary artery disease. LA mechanics and left ventricular global longitudinal strain (LV-GLS) were assessed by STE. The cohort was followed-up for a median of 30 (IQR 12.6–50) months, and outcomes were determined (combined outcome of HF, death, and aortic valve replacement).

**Results:**

In our sample set, values of LV-GLS (− 18.5% vs − 17.1, *p* = 0.025), E/e’ ratio (15.8 vs 18.4, *p* = 0.03), and global LA mechanics (LA ɛsys, 23% vs 13.8%, *p* < 0.001) were worse for sAS compared to those for mAS. However, LA ɛsys (AUC 0.85, 95% CI 0.78–0.90, *p* < 0.001), ɛe (AUC 0.83, 95% CI 0.75–0.88, *p* < 0.001), and ɛa (AUC 0.80, 95% CI 0.70–0.84, *p* < 0.001) were the best discriminators of sAS, with sensitivities higher than 85%. LA ɛsys showed a stronger correlation with both aortic valve area (*r*^*2*^ = 0.6, *p* < 0.001) and mean LV/aortic gradient (*r*^*2*^ = 0.55, *p* < 0.001) than LV-GLS (*r*^*2*^ = 0.3 and *r*^*2*^ = 0.25, *p* = 0.01). Either LV-GLS or LA ɛsys, but not the E/e’ ratio, TAPSE, or RV/RA gradient, were a significant predictors of the combined outcome.

**Conclusions:**

LA global strain was the best discriminator of severity, surpassing E/e’ ratio and LV-GLS, and a significant predictor of prognosis in AS.

## Background

Aortic valve stenosis (AS) is currently the most common valvular heart disease, and its prevalence is increasing as the population ages [1]. Currently, the management of patients with AS is based on the assessment of AS severity, left ventricular ejection fraction (LVEF), and symptom development [2]. In patients with aortic valve disease, the left atrium (LA) undergoes remodeling due to pressure overload, resulting in disturbances in three functional phases: reservoir, conduit, and contractile phase [3, 4]. In patients with AS, there is an increase in filling pressures and LA afterload, due to left ventricular (LV) hypertrophy. The increase in LA afterload affects its triphasic function, with particular loss of LA contractile function [5]. Reservoir and conduit phases damage is less evident and probably occurs in more advanced states, related to pulmonary hypertension [5, 6].

Speckle-tracking echocardiographic (STE) analysis allows a rapid and practical assessment of the atrial deformation profile, due to its semiautomated system and offline processing [4, 7].

LA mechanics assessed by STE have been studied for cardiovascular disease in different clinical settings [4]. LA strain is a prognostic marker for mitral valvulopathy [6] and is correlated with pulmonary hypertension in patients with severe AS (sAS) [6]. Moreover, in sAS, atrial function is an independent predictor of postoperative atrial fibrillation (AF) in patients undergoing aortic valve replacement (AVR) [8]. Preoperative early mitral inflow velocity-to-early diastolic strain rate (E/SRe) ratio was significantly associated with long-term postoperative survival and was superior to the E/e’ ratio in patients with sAS undergoing AVR [9].

However, there is limited information on LA mechanics in AS and how they vary according to the severity of the disease. Elucidation of independently associated parameters of severity that can aid in the diagnosis of AS and determining the need for AVR in doubtful cases (e.g., paradoxical AS) is clinically relevant. Moreover, it is pertinent to understand the mechanisms responsible for poor prognosis or suboptimal results in patients undergoing AVR.

The aim of our study was to analyze LA mechanics through STE in AS to find better discriminators of disease severity and prognosis, beyond classic echocardiographic parameters. We also sought to correlate LA mechanics to known markers of severity, such as aortic valve area (AVA) and mean LV/aortic gradient.

## Methods

### Study population

We conducted a retrospective analysis of a prospectively enrolled cohort of 102 patients diagnosed with sAS and 80 patients with moderate AS (mAS). Patients with heart surgery, coronary lesions, segmental wall-motion abnormalities, hypothyroidism, LVEF < 50%, and/or poor acoustic window were excluded. Importantly, we also have excluded patients with paradoxical low flow low gradient aortic stenosis [this is, an AVA ≤1.0 cm2 or indexed AVA ≤0.6 cm2/m2, a mean pressure gradient (MPG) < 40 mmHg, a LVEF ≥50% and a stroke volume index (SVi) < 35 mL/m2].

The study was approved by the institutional scientific and bioethical committees and was performed in accordance with the Declaration of Helsinki.

### Study procedures

We analyzed the epidemiologic, clinical, analytical, and echocardiographic data (namely, 2D-STE global longitudinal strain (GLS) analysis) of the selected population (patients with sAS and mAS). The cohort was followed-up during a median period of 30 months (IQR 12.6–50), and outcomes (hospital admission for heart failure (HF), death, and AVR through surgery or percutaneously) were determined.

Preliminary data was presented by the authors at Poster Session European Heart Journal - Cardiovascular Imaging, January 2019 [10].

### Echocardiographic data

Echocardiographic examination included tissue Doppler imaging (TDI) and STE analysis of LV, LA, and right ventricular (RV) functions, as previously described [6, 11]. We used a Vivid 7 (GE Healthcare, Horten, Norway) cardiovascular (CV) ultrasound device, with a 1.7/3.4-MHz tissue harmonic transducer. Standard echocardiographic views where obtained with 60–80 fps in 2D imaging. Echocardiographic data were analyzed offline using a specific software (EchoPAC 16.0, GE Healthcare, Horten, Norway).

### AS severity

We measured aortic transvalvular peak velocities through continuous-wave Doppler, obtained peak and mean from the simplified Bernoulli equation and aortic valve area through the continuity solution equation [12].

### Left ventricular dimensions and function

We followed to the current recommendations [13, 14] to measure LV size and systolic and diastolic functions. Peak LV-GLS was assessed by STE using a 16-segment model [11, 15].

### LA dimensions and function

Analysis of LA deformation by STE was performed on four-chamber, with three consecutive heart cycles being recorded during breath hold and a frame rate of 60–80 fps, as recommended. Automatic offline software analysis generated and averaged strain curves for each atrial segment [16]. P-wave onset marked the initial frame of processing. LA global strain and strain rate during systole (LA ɛsys and SRs), early diastole (LA ɛe, SRe), and late diastole (LA ɛa, SRa) corresponding to the LA reservoir, conduit, and contractile functions, respectively, were measured [4].

## Statistical analysis

Normality of continuous variables was assessed by histogram observation and the Kolmogorov–Smirnov test. Continuous variables were expressed as mean ± standard deviation and categorical variables as percentage. Student’s t-test or ANOVA was used for group comparisons. Individual variables were assessed for homogeneity of variance using Levene’s test. For categorical variables, the chi-square or Fisher’s exact test was used, as appropriate.

A receiver operating characteristic (ROC) curve analysis was performed to compute the discriminative power of LA mechanics, LV 2D-STE, E/e’ ratio, tricuspid annular plane systolic excursion (TAPSE), or RV/right atrium (RV/RA) gradient in sAS and mAS. A comparison of ROC curves was executed using the Delong method.

Relationships between different parameters were assessed by correlation analysis: Pearson’s method for continuous, normally distributed variables and Spearman’s method for continuous but skewed variables.

Survival analysis was performed using Kaplan-Meier curves, with the date of entry into the study defined as the date of the diagnosis (first echocardiography). Patients that did not die were censored at the end of the study.

Univariate Cox’s proportional hazards analysis was used to to identify independent predictors of outcomes in the overall AS population.

A *P*-value (two-sided) < 0.05 indicated statistical significance. Stata (Stata IC for Windows, version 13, Lakeway Drive, TX, USA) and MedCalc statistical software (MedCalc software for Windows, version 14.8.1, Ostend, Belgium) were used for the statistical analyses.

## Results

### Study population

The clinical and echocardiographic features are shown in Table 1. The mean patient age was 76 ± 7.9 years, and 51% of the patients were male. The mean values were as follows: 17.7 ± 3.9% for LV-GLS, 41 ± 12.1 mL.m^− 2^ for indexed LA volume (LAVI), 17.2 ± 2% for E/e’ ratio, 20.3 ± 3.5 mm for TAPSE, and 24.1 ± 10 mmHg for RV/RA gradient. The mean aortic valve area (AVA) was 0.9 ± 0.3 cm^2^, and LV/aortic gradient was 40.7 ± 12.8 mmHg.

**Table 1:** Clinical and echocardiographic data of the study population

	sAS	mAS	*P-value*
Age (±SD, years)	76.4 (±8.8)	76.2 (±6.9)	0.834
Men (%)	54.9	46.9	0.337
Atrial fibrillation (%)	24.4	25	0.932
Symptoms (%)	86.6	21.9	< 0.001
LVEF (±SD, %)	60.3 (±7.3)	62.2 (±5.4)	0.084
LVDD (±SD, mm)	51.6 (±6.7)	52.2 (±7.4)	0.604
LVSD (±SD, mm)	33.8 (±7.1)	32.6 (±6.7)	0.238
IVS (±SD, mm)	12.8 (±3.8)	11.5(±2.3)	0.021
E/e’ (±SD)	18.4 (±8.0)	15.8 (±4.3)	0.034
TAPSE (±SD, mm)	20.2 (±3.6)	20.6 (±3.3)	0.488
PASP (±SD, mmHg)	28.9 (±11.8)	29.5 (±10.1)	0.779
LAVI (±SD, mL.m^−2^)	42.6 (±12.3)	39.5 (±12.2)	0.129
LV-GLS (±SD,%)	−17.1 (±3.84)	−18.5 (±3.85)	0.025
LA ɛsys (±SD,%)	13.8 (±5.7)	23.1 (±7.0)	< 0.001
LA ɛe(±SD,%)	6.5 (±3.2)	11.5(±4.4)	< 0.001
LA ɛa(±SD,%)	7.1(±3.9)	11.5(±4.4)	< 0.001
LA SRs(±SD,%)	0.8 (±0.3)	1.0(±0.3)	< 0.001
LA SRe(±SD,%)	−0.5(±0.3)	−0.7(±0.3)	0.003
LA SRa(±SD,%)	−1.03 (±0.4)	−1.2(±0.4)	0.017
Global strain (±SD,%)	4.6(±0.9)	−3.1 (±0.7)	< 0.001

*IVS* interventricular septum, *LAVI* left atrial volume (indexed), *LA ɛsys* left atrial systolic strain (reservoir function), *LA ɛe* left atrial early diastolic strain (conduit function), *LA ɛa* left atrial late diastolic strain (contractile function), *LA SRs* left atrial systolic strain rate (reservoir function), *LA SRe* left atrial early diastolic strain rate (conduit function), *LA SRa* left atrial late diastolic strain (contractile function), *LV-GLS* left ventricular global longitudinal strain, *LVDD* left ventricular end-diastolic diameter, *LVSD* left ventricular end-systolic diameter, *LVEF* left ventricular ejection fraction, *mAS* moderate aortic stenosis, *PASP* pulmonary artery systolic pressure, *sAS* severe aortic stenosis, *TAPSE* tricuspid annular plane systolic excursion.

### STE-GLS and LA mechanics analysis in AS

Among classic echocardiographic parameters, only interventricular septum (IVS) diameter (12.8 vs 11.5 mm, *p* = 0.021) and E/e’ ratio (15.8 vs 18.4, *p* = 0.03) had worse values in sAS compared to that in mAS. On 2D-STE analysis, LV-GLS (− 18.5% vs − 17.1, *p* = 0.025) and LA global mechanics (LA ɛsys, 23% vs 13.8%, *p* < 0.001) were more impaired in sAS. Global strain (the sum of LV-GLS and reservoir LA strain [LA ɛsys]) had negative values and was statistically lower in sAS (*p* < 0.001).

**Fig. 1:**
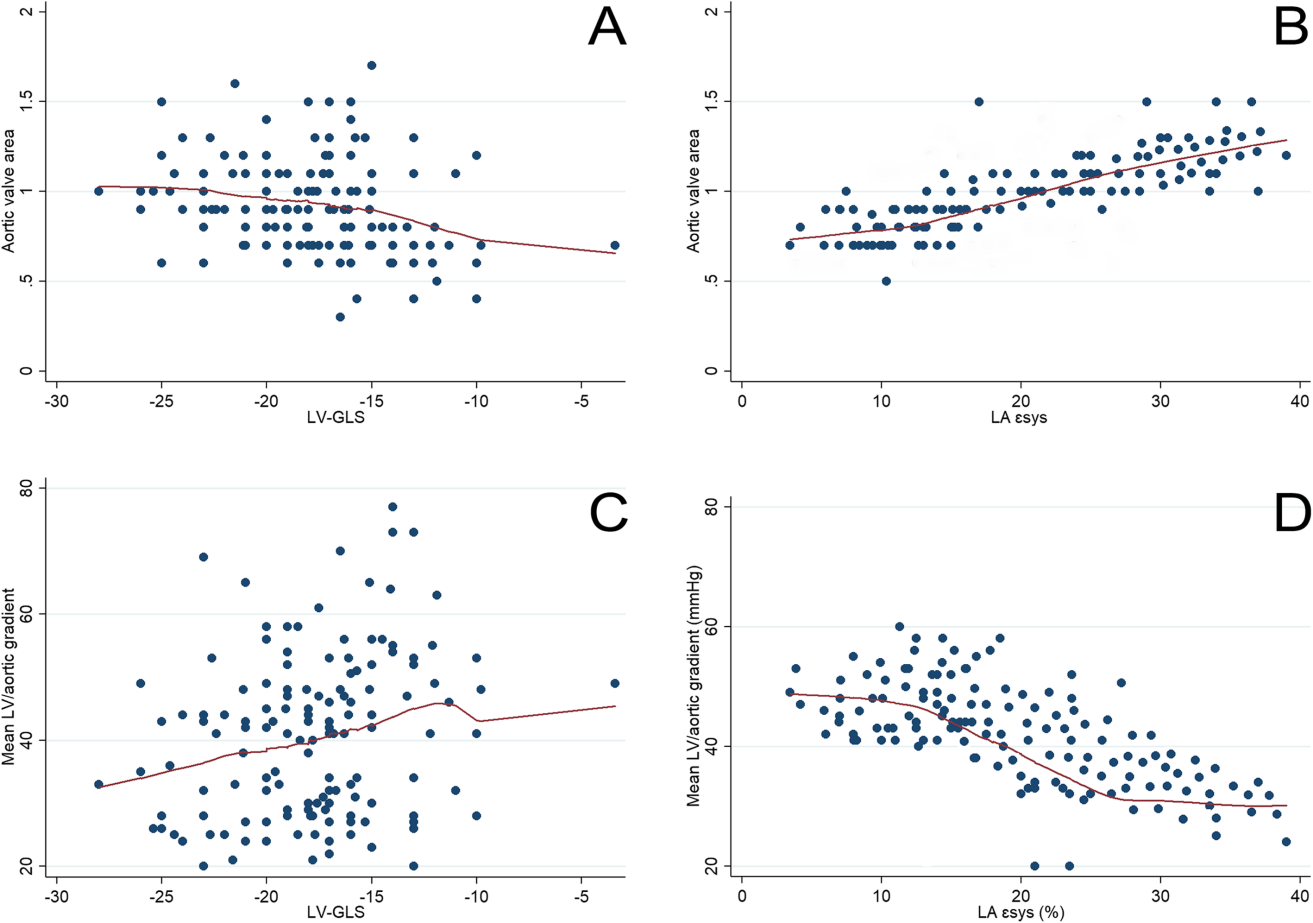
Linear regression analysis of LV-GLS with aortic valve area (**a**) versus LA ɛsys with aortic valve area (**b**); LV-GLS with mean LV/aortic gradient (**c**) versus LA ɛsys LV/aortic gradient (**d**). GLS, global longitudinal strain; LA, left atrium; LV, left ventricle

Correlation analysis of classic parameters and STE (LA vs LV) showed that LA ɛsys was closely related to both aortic valve area (*r*^*2*^ = 0.6, *p* < 0.001) and mean LV/aortic gradient (*r*^*2*^ = 0.55, *p* < 0.001), when comparing LV-GLS to these two parameters (*r*^*2*^ = 0.3 and *r*^*2*^ = 0.25, *p* = 0.01) (Fig. 1).

### Discriminators of AS severity

**Fig. 2:**
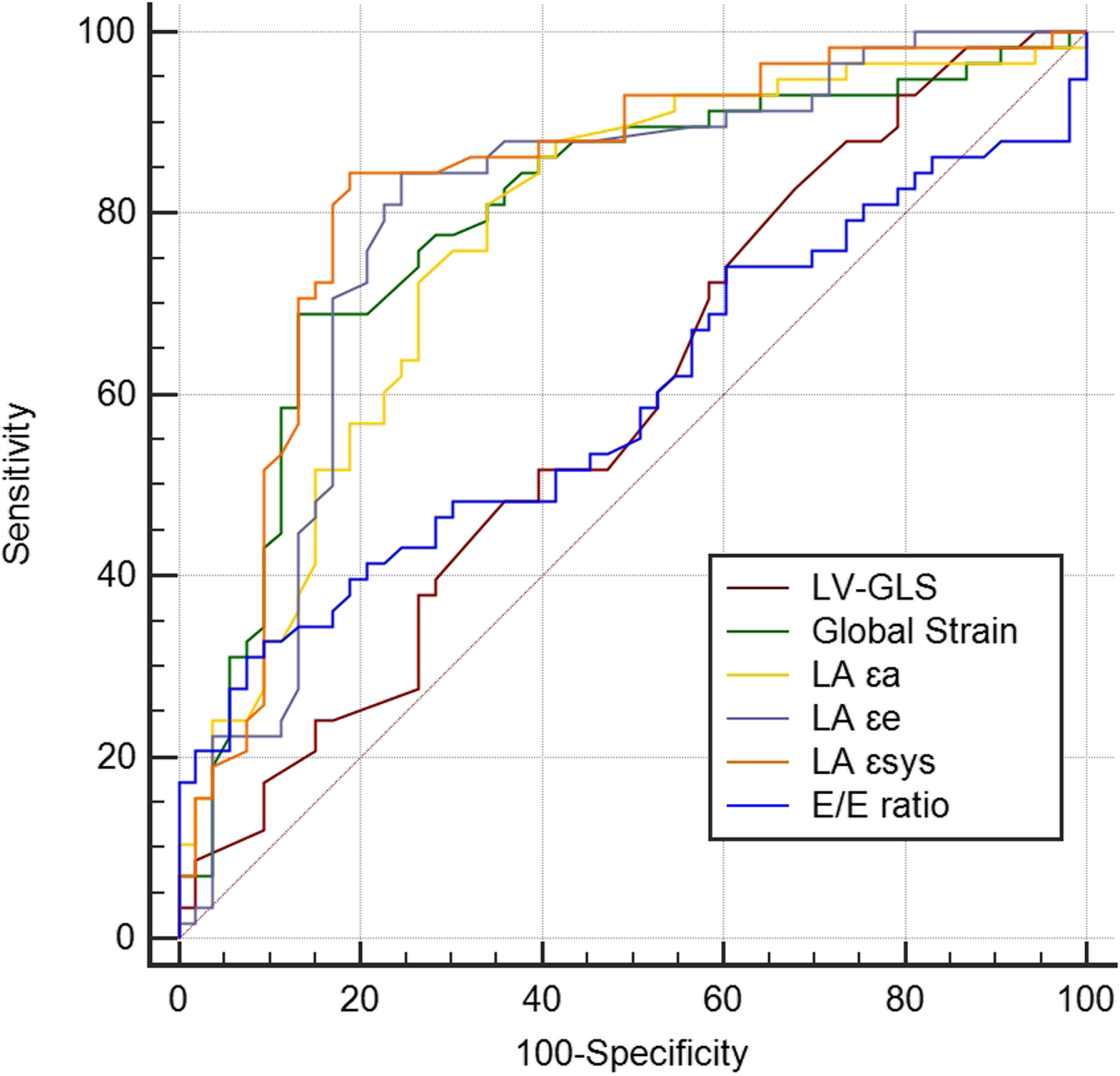
ROC analysis curves for discriminating mAS versus sAS. LA mechanics was a better discriminator of AS severity. LV-GLS vs global strain, *P* = 0.009; LV-GLS vs LA ɛa, *P* = 0.004; LV-GLS vs LA ɛe, *P* = 0.001; LV-GLS vs LA ɛsys, *P* < 0.001; global strain vs E/e’ ratio, *p* = 0.001; LA ɛa vs LA ɛsys, *P* = 0.038; LA ɛa vs E/e’ ratio, *P* = 0.003; LA ɛe vs E/e’ ratio, *P* = 0.001; LA ɛsys vs E/e’ ratio, *P* < 0.001; LV-GLS vs E/e’ ratio, NS; global strain vs LA ɛe, NS; global strain vs LA ɛa, NS; global strain vs LA ɛsys, NS; LA ɛa vs LA ɛe, NS; LA ɛe vs LA ɛsys, NS. LA ɛsys, left atrial systolic strain (reservoir function); LA ɛe, left atrial early diastolic strain (conduit function); LA ɛa, left atrial late diastolic strain (contractile function); LV-GLS, left ventricular global longitudinal strain; mAS, moderate aortic stenosis; sAS, severe aortic stenosis

Compared to classic echocardiographic parameters and even LV-GLS, LA strain parameters emerged as the best discriminators of AS severity, with mean AUCs of 0.8 or more and sensitivities higher than 85%. Global strain also had an AUC above 0.8 and was the most specific factor for sAS (Table 2 and Fig. 2).

**Table 2:** Discriminative power of echocardiographic parameters according to aortic stenosis severity

	AUC	95% CI	*P-value*	Sensitivity (%)	Specificity (%)	Criterion
LA ɛsys (%)	0.870	0.799–0.923	< 0.001	85.0	64.1	11
LA ɛe (%)	0.824	0.747–0.886	< 0.001	88.7	73.4	9.4
LA ɛa (%)	0.810	0.732–0.874	< 0.001	86.6	76.6	18.5
Global strain (%)	0.809	0.735–0.870	< 0.001	69.6	85.9	0.56
LA SRs (%)	0.707	0.626–0.779	< 0.001	41.5	90.6	0.64
LA SRe (%)	0.645	0.556–0.728	0.001	71.9	56.1	0.28
LA SRa (%)	0.628	0.538–0.712	0.006	50.7	75.0	−1
IVS (mm)	0.623	0.540–0.702	0.001	46.3	76.6	12
LV-GLS (%)	0.606	0.516–0.691	0.056	77.2	37.5	−20
LAVI (mL.m^−2^)	0.593	0.508–0.675	0.054	59.8	62.5	39.7
E/e’ ratio	0.588	0.493–0.678	0.097	31.8	90.6	21.5
TAPSE (mm)	0.543	0.458–0.627	0.427	42.5	75	18
PASP (mmHg)	0.531	0.446–0.615	0.517	41.8	73.4	23

*AUC* area under the curve, *IVS* interventricular septum, *LAVI* left atrial volume (indexed), *LA ɛsys* left atrial systolic strain (reservoir function), *LA ɛe* left atrial early diastolic strain (conduit function), *LA ɛa* left atrial late diastolic strain (contractile function), *LA SRs* left atrial systolic strain rate (reservoir function), *LA SRe* left atrial early diastolic strain rate (conduit function), *LA SRa* left atrial late diastolic strain (contractile function), *LV-GLS* left ventricular global longitudinal strain, *PASP* pulmonary artery systolic pressure, *TAPSE* tricuspid annular plane systolic excursion

Table 3 shows a schematic redistribution of AS severity according to different LA strain parameters, in which 5% (LA ɛa) to 30%(global strain) cases of moderate AS have criteria of severity. In classic severe AS, the majority of cases have severity criteria, except when based on LAɛsys, where 69% cases have preserved values of this parameter.

**Table 3:** Reclassification of aortic stenosis severity based on different severity parameters

Mean Gradient (mmHg)	Moderate AS (*n=80*)		Severe AS (*n=102*)		Severity criterion > 40
	80		102		
LA ɛsys (%)	≥11	< 11	≥11	< 11	Severity criterion < 11
	75	5	70	32	
LA ɛe (%)	≥9.4	< 9.4	≥9.4	< 9.4	Severity criterion < 9.4
	59	21	16	86	
LA ɛa (%)	≥18.5	< 18.5	≥18.5	< 18.5	Severity criterion < 18.5
	76	4	4	98	
Global strain (%)	≥0.56	< 0.56	≥ 0.56	< 0.56	Severity criterion < 0.56
	56	24	25	77	

*LA ɛsys* left atrial systolic strain (reservoir function), *LA ɛe* left atrial early diastolic strain (conduit function), *LA ɛa* left atrial late diastolic strain (contractile function)

### AF

The prevalence of AF in our cohort was 24.4% for sAS and 25% for mAS. Although there was no statistically significant difference in prevalence between the two groups, we decided to perform a multivariate regression model. We analyzed the influence of AF in echocardiographic parameters, particularly LV and atrial strain analysis, according to the severity of AS (Table 4). Although having more influence in worse LV-GLS values (AF impaired LV-GLS by an order of 0.9%, while sAS by 0.48%), AF had lower impact on LA mechanics (impairing LA ɛsys by 3.55% and LA ɛe by 2.6%, while sAS impaired LA ɛsys by 8.31%, LA ɛe by 3.9%, and LA ɛa by 3.5%; all *p* < 0.001).

**Table 4:** Multivariate regression model for discriminating the effect of the severity of AS versus atrial fibrillation by several strain parameters

Multivariate regression model	β-coefficient	95% CI	*P-value*
LV-GLS (%)			
sAS	1.48	0.2; 2.7	0.021
AF	1.9	0.4; 3.3	0.012
LA ɛsys (%)			
sAS	−9.31	−11.3; −7.3	< 0.001
AF	−4.55	−6.8; −2.3	< 0.001
LA ɛe (%)			
sAS	−4.9	−6.22; −3.7	< 0.001
AF	−0.9	− 2.3; 0.6	0.248
LA ɛa (%)			
sAS	−4.5	−5.7; − 3.2	< 0.001
AF	−3.6	−5.1; −2.1	< 0.001

*AF* atrial fibrillation, *LA ɛsys* left atrial systolic strain (reservoir function), *LA ɛe* left atrial early diastolic strain (conduit function), *LA ɛa* left atrial late diastolic strain (contractile function), *LA SRs* left atrial systolic strain rate (reservoir function), *LA SRe* left atrial early diastolic strain rate (conduit function), *LA SRa* left atrial late diastolic strain (contractile function), *LV-GLS* left ventricular global longitudinal strain, *sAS* severe aortic stenosis

### Survival and event-free rate analysis

**Fig. 3:**
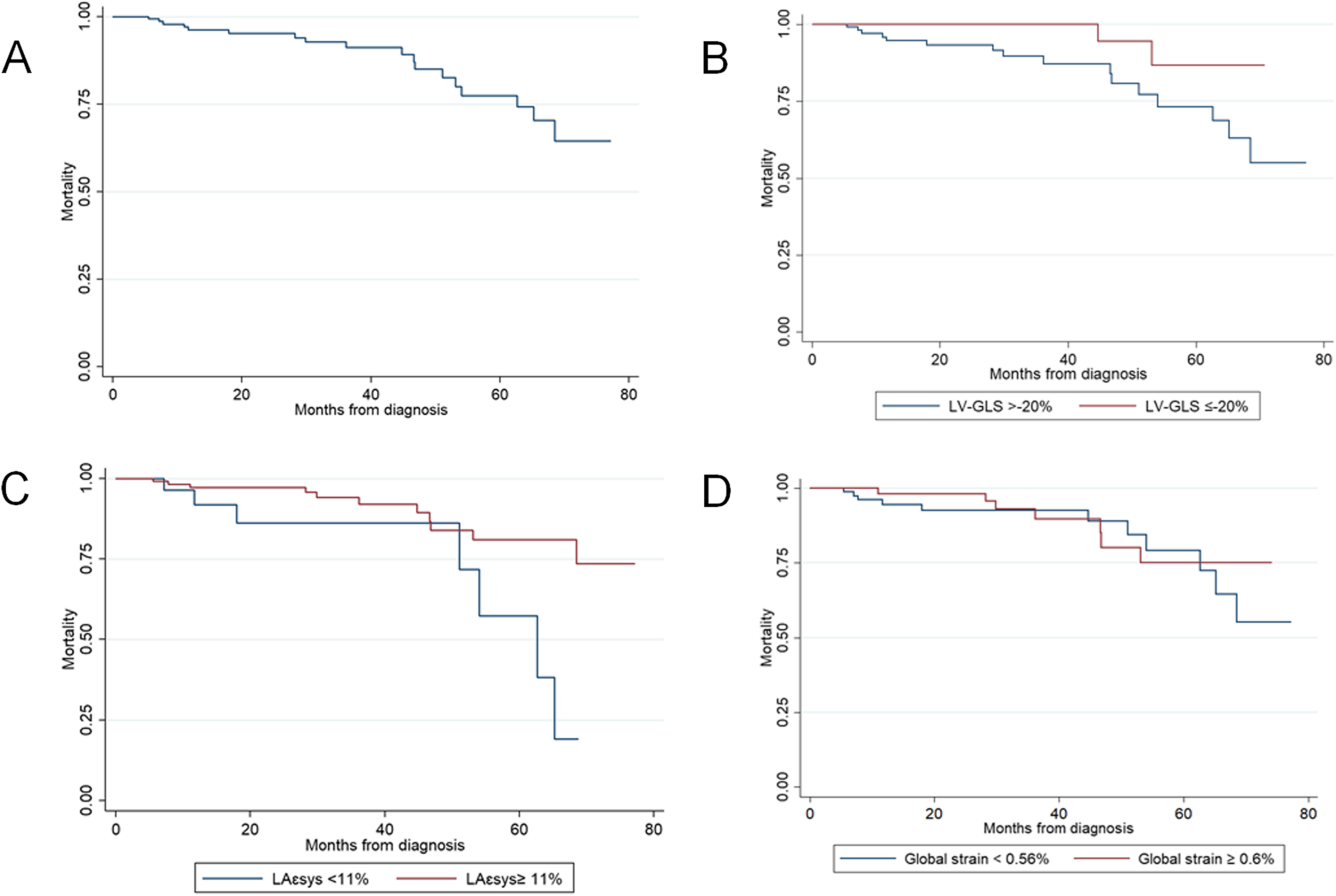
Kaplan-Meier survival curves in the general cohort (**a**), according to LV-GLS (**b**), LA ɛsys, (**c**) and global strain (**d**). LA ɛsys, left atrial systolic strain (reservoir function); LV-GLS, left ventricular global longitudinal strain

Kaplan-Meier curves are depicted in Fig. 3. Results of the Cox regression analysis is presented on Table 5. Only LA-SRS and GLS were significant predictors of HF (BNP, TAPSE, E/e’ ratio, AF, RV/RA gradient were not). AVR was predicted by mean gradient, AVA, LA ɛsys and LAɛe. Predictors of death were age, BNP, LA ɛe and GLS. Regarding the combined outcome of HF, death, and AVR, LV-GLS, LA ɛsys and global strain, but not E/e’ ratio, TAPSE or RV/RA gradient, were significantly associated with poor outcomes.

**Table 5:** Cox regression analysis results

Outcomes	Predictors	HR (95%CI)	*P value*
Heart failure	LA-SRs	0.17 (0.1-0.8)	0.022
	GLS	1.11 (1.0-1.3)	0.045
	BNP	1.0 (0.9-1.01)	0.810
	E/e’ ratio	1.0 (0.9-1.1)	0.710
	TAPSE	0.96 (0.84-1.1)	0.522
	RV/RA gradient	1.0 (0.9-1.1)	0.667
Aortic valve replacement	Mean gradient	1.40 (1.1-1.6)	< 0.001
	AVA	0.04 (0.01-0.14)	< 0.001
	LA ɛsys	0.95 (0.91-0.99)	0.027
	LA ɛe	0.91 (0.85-0.98)	0.026
Death	BNP	1.2 (1.0-1.3)	0.002
	LA ɛe	0.84 (0.74-0.96)	0.010
	GLS	1.22 (1.08-1.41)	0.003
Combined outcome	LV-GLS (%)	1.16 (1.08-1.23)	< 0.001
	LA ɛsys (%)	0.9 (0.92-0.93)	< 0.001
	Global strain	0.95 (0.93-0.98)	0.035
	E/e’ ratio	1.01 (0.97-1.04)	0.667
	TAPSE (mm)	0.97 (0.01-1.03)	0.414
	RV/RA gradient	1.01 (0.98-1.04)	0.285

*LA ɛsys* left atrial systolic strain (reservoir function), *LA ɛe* left atrial early diastolic strain (conduit function), *LA ɛa* left atrial late diastolic strain (contractile function), *LA SRs* left atrial systolic strain rate (reservoir function), *LA SRe* left atrial early diastolic strain rate (conduit function), *LA SRa* left atrial late diastolic strain (contractile function), *LV-GLS* left ventricular global longitudinal strain, *RV/RA gradient* right ventricle/right atrium gradient, *TAPSE* tricuspid annular plane systolic excursion

## Discussion

We described the LA function in patients with stenotic aortic valve disease and assessed its impact on severity and prognosis. In our study, 2D-STE LV-GLS and global LA mechanics were more impaired in sAS. LA ɛsys was closely related to both aortic valve area and mean LV/aortic gradient when compared to LV-GLS. Moreover, LA strain parameters were the best discriminators of AS severity, with mean AUCs of 0.8 or more and sensitivities higher than 85%. Regarding prognosis, LV-GLS, LA ɛsys, and global strain were better correlated with the combined outcome of HF, death, and AVR.

AS causes LV remodeling with decreased LV compliance, increased diastolic pressure and LA afterload. In earlier stages, LA preload is normal and augments with LA volume [5]. In our study, volumetric parameters did not vary according to AS severity (mAS vs sAS), while LV filling pressures (E/e’ ratio) did. This shows a gradual increase in diastolic dysfunction, consistent to the severity of AS.

LA mechanics assessment was performed through STE, which, by allowing selective analysis of myocardial layers (when compared to TDI), guarantees an optimized analysis of the LA thin myocardial layer [5, 17].

We demonstrated that in patients with AS, the LA ɛsys was closely associated with both the aortic valve area and mean LV/aortic gradient, while LV-GLS was not. This can be indicative of impairment of LA compliance, even before the onset of LV subendocardial dysfunction in aortic valvular disease [5, 6].

IVS, E/e’ ratio, LV-GLS, and LA mechanics were significantly impaired in sAS. Among these, LA mechanics were strongly associated with severity: LA ɛsys (reservoir) had the highest AUC, and LA ɛe (conduit) had the highest sensitivity, while global strain and LA ɛa (contractile) had the highest specificities.

Moderate valvular disease shows only impaired values for LA function in the contractile phase, with normal values for the reservoir and conduit phases [5]. This may be due to an increase in LA afterload, resulting in atrial myofibril damage and contractile dysfunction. In the initial stages of LA remodeling, the interstitial collagen deposition is not extensive; therefore, LA compliance is preserved. This may be because in our cohort, the most discriminative parameter for sAS was LA ɛsys (reservoir), with global strain being the most specific parameter.

Strain analysis allowed rearrangement of AS cases according to severity criteria. We could find 5 to 30% cases of moderate AS that had severity criteria (Table 3). This distribution in the severe AS cohort was less accurate with a somewhat heterogenous distribution.

When assessing prognosis, LV-GLS, LA ɛsys, and global strain emerged as significant predictors of the combined outcome compared to the classic parameters, such as E/e’ ratio, TAPSE, or RV/RA gradient.

Previous studies have shown that LA reservoir function is associated with a poor prognosis in the general population and in patients with AF and mitral stenosis [18, 19]. Also, it has been previously reported that LA ɛsys is a strong predictor of major adverse cardiac events, as also the functional class and coronary artery disease, in patients with sAS [20]. In our study, we assessed LA mechanics not only in sAS, but also in mAS, and LA ɛsys was associated with worse outcomes in both groups. Similarly, we excluded the presence of coronary artery disease, precisely because it would interfere in the analysis of discriminators of severity and worse prognosis.

Monitoring LA function in patients with AS can provide valuable information. First, LA mechanics has a greater discriminative power than other classic echocardiographic parameters for assessing severity and is closely associated with classic measures, such as mean LV/aortic gradient. Thus, LA mechanics can provide additional diagnostic information in doubtful cases, such as paradoxical low-flow and low-gradient AS. Second, LA mechanics was associated with worse outcomes, therefore can enhance prognosis assessment and help better define the appropriate surgical or percutaneous intervention timing in doubtful cases.

## Limitations

Although both groups of patients were moderately represented, and the sample size was suitable for data analysis, these findings must be conformed in a larger population with longitudinal studies.

## Conclusions

This study reports that LA-GLS can be a useful tool to better determine severity in AS. Compared to classic parameters, such as E/e’ ratio, LA mechanics are more closely associated with mean LV/aortic gradient and valve area. Moreover, LA mechanics and LV-GLS provide valuable information for assessing prognosis in patients with AS. These data can be useful in clinical practice for severity calculation and prognostic evaluation, such as decision and timing of AVR, when evaluating doubtful cases.

## Data Availability

The datasets used and/or analyzed during the current study are available from the corresponding author on reasonable request.

## Abbreviations

AF: Atrial fibrillation
AS: Aortic stenosis
AVR: Aortic valve replacement
CI: Confidence interval
CV: Cardiovascular
HF: Heart failure
IVS: Interventricular septum
LA ɛa: Left atrial late diastolic strain (contractile function)
LA ɛe: Left atrial early diastolic strain (conduit function)
LA ɛsys: Left atrial systolic strain (reservoir function)
LA SRa: Left atrial late diastolic strain (contractile function)
LA SRe: Left atrial early diastolic strain rate (conduit function)
LA SRs: Left atrial systolic strain rate (reservoir function)
LA: Left atrium
LAVI: Left atrial volume (indexed)
LVDD: Left ventricular end-diastolic diameter
LVEF: Left ventricular ejection fraction
LV-GLS: Left ventricular global longitudinal strain
LVSD: Left ventricular end-systolic diameter
mAS: Moderate aortic stenosis
PASP: Pulmonary artery systolic pressure
RA: Right atrium
ROC: Receiver operating characteristic
RV: Right ventricle
sAS: Severe aortic stenosis
STE: Speckle-tracking echocardiography
TAPSE: Tricuspid annular plane systolic excursion
TTE: Transthoracic echocardiography
